# GC/MS profiling of essential oils from *Bontia daphnoides* L., chemometric discrimination, isolation of dehydroepingaione and evaluation of antiviral activity

**DOI:** 10.1038/s41598-022-22174-4

**Published:** 2022-10-21

**Authors:** Amany A. Thabet, Saad Moghannem, Iriny M. Ayoub, Fadia S. Youssef, Eman Al Sayed, Abdel Nasser B. Singab

**Affiliations:** 1 Faculty of Pharmacy, Department of Pharmacognosy, Ain Shams University, Abbassia, Cairo, 11566 Egypt; 2 Department of Botany and Microbiology, Faculty of Science, Al-Azhar University, Nasr City, Cairo, Egypt; 3 Center for Drug Discovery Research and Development, Faculty of Pharmacy, Ain Shams University, Cairo, 11566 Egypt

**Keywords:** Natural products, Chromatography, Molecular modelling, Viral infection

## Abstract

*Bontia daphnoides* L. has been utilized in traditional medicine for treatment of herpes, cough and colds. The aim of this study was to analyze the volatile constituents of this plant by GC/MS (Gas Chromatography coupled to Mass Spectrometry) and to assess their antiviral activity. A total of 64 compounds were identified where dehydroepingaione represented 83.60, 72.36, 58.78 and 34.18% in the leaves, stems, flowers and fruits, respectively. Principal component and hierarchical cluster analysis revealed the discrimination of the organs as the leaves and stems were distributed in the same cluster in contrast to the flowers and fruits. Furthermore, the antiviral activity was assessed where the oils of leaves and stems exhibited potent antiviral activity displaying IC_50_ of 11.98, 12.62 µg/ml against HSV-1 and 13.34, 14.50 µg/ml against CoxB4, respectively. Dehydroepingaione was isolated from the *n*-hexane fraction of the leaves and showed activity against HSV-1 and CoxB with IC_50_ of 24.46 and 25.32 µg/ml, respectively. Molecular modelling studies illustrated that the major compounds showed good affinity towards HSV type-1 thymidine kinase. Therefore, it can be concluded that the oils from *B. daphnoides* have promising antiviral activity that may be attributed to the major oxygenated sesquiterpenes.

## Introduction

Viral diseases represent serious and challenging problems that affect human’s health. The difficulty of viral infections is principally due to the insufficiency of antiviral agents, the ability of viruses to produce drug-resistant mutants and the lack of a vaccine or its resistance^1^. The mechanism of action of antiviral drugs may involve preventing viral attachment to host cells, inhibiting protease, preventing uncoating, stopping nucleic acid synthesis and hindering viral release^2^. Coronavirus disease 2019 (COVID-19) pandemic was responsible for high intensive care unit (ICU) admission rates and high mortality worldwide^3^. Therefore, discovering new drug entities with antiviral activity has become crucial to combat and overcome the problems of viral infections^2^. Herpes simplex virus type 1(HSV-1) is a common infection that causes cold sores and affects nearly 48% of people. It may cause complications mainly in immune-compromised patients^1^. The approved drug for treatment of HSV-1 is acyclovir, however, the development of drug resistant mutants have made the necessity for the search for new antiviral agents^1,4^. Coxsackie B viruses have been reported to have high incidence in Children. Their main clinical manifestations are sore throat with an abrupt onset of fever, dysphagia, malaise and myocarditis^5^. Infection by Coxsackie B virus type 4 (CoxB4) has been linked to insulin-dependent diabetes mellitus. No specific antiviral agents was found against CoxB4^4^.

Medicinal plants have been traditionally known as a source of bioactive compounds with antimicrobial activity. They could offer alternatives to synthetic traditionally known antiviral drugs with new mechanisms, more efficacy, lower toxicity, better acceptability and lower cost^2,4,6^. Scrophulariaceae Juss. is a family of annual and perennial herbs that consists of approximately 74 genera and 1533 species broadly dispersed around the world^7–9^. The present concept of Scrophulariaceae includes at least eight major tribes: Aptosimeae, Buddlejeae, Hemimerideae, Leucophylleae, Limoselleae, Myoporeae, Scrophularieae, and Teedieae^10,11^. The members of the tribe Myoporeae have antiviral, antibacterial, antifungal and insecticidal activities, also they produce essential oil rich in sesquiterpenes and monoterpenes^12^.

*Bontia* is a monotypic genus in the tribe Myoporeae restricted to the West Indies and is known as, olive bush and kidney bush^13,14^. It was traditionally used as leaf infusion for the treatment of diabetes, jaundice, nephritis, hypertension, cough and colds^14–16^. Extracts of the plant were used to control intestinal worms, insect bites, herpes, inflammation, ulcers and wounds. Leaves were steeped and the brew was administered to people suffering from fish poisoning^16^. Few studies were reported on the chemical constituents and biological activities of *Bontia daphnoides.* An insecticidal compound, epingaione with LC_50_ 20.8 (μg/insect), was isolated from the leaves of *B. daphnoides*^17^. Furthermore, the same compound exhibited 79.2 and 50.8% antiproliferation/cytotoxic activity on the human SH-SY5Y neuroblastoma and TE-671 sarcoma cells in vitro at 50 µg/mL, respectively^14^.

No previous reports were found on the chemistry and biology of the essential oil of this plant. Therefore, our aim in this study was to determine the chemical composition of the volatile constituents of *B. daphnoides* leaves, stems, flowers and fruits by GC/MS. Also, chemometric analysis implying Principle Component Analysis (PCA) and Hierarchical Cluster Analysis (HCA) was performed for discrimination of the different organs using the obtained GC/MS data. Moreover, the antiviral activity of the isolated oils was assessed against HSV-1 and CoxB4. In addition, a molecular modelling study was implemented for the major identified compounds on the active sites of HSV type-1 thymidine kinase (TK) and HSV type-1 DNA polymerase (DP) that are commonly used as molecular targets for antiviral drugs. This could help to understand the antiviral potential of the volatile constituents of different organs of *B. daphnoides* for developing a new antiviral drug from these oils.

## Results

### GC/MS analyses of B. daphnoides essential oils

**Figure 1:**
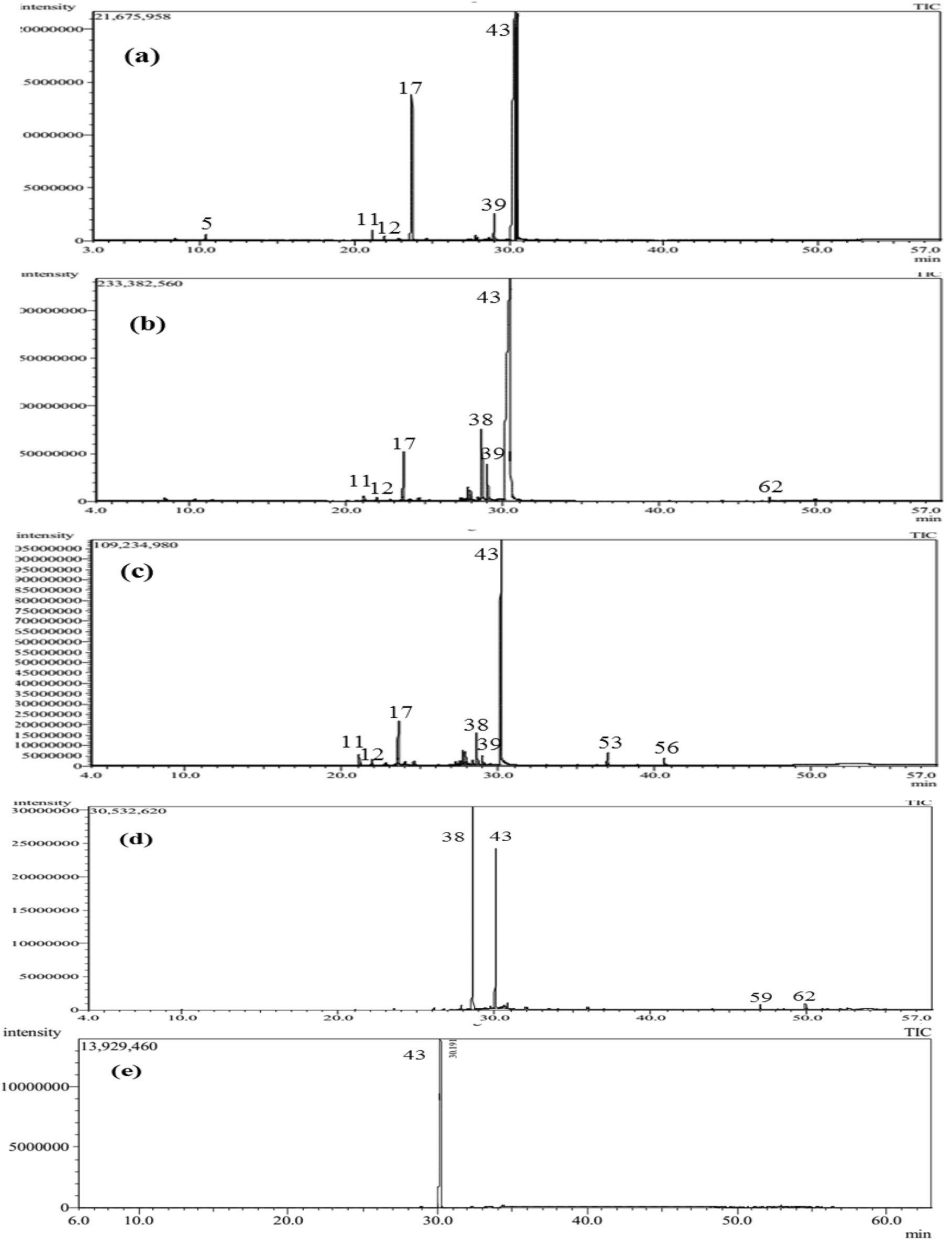
GC-chromatograms obtained with a Rtx-5MS column of the volatile constituents isolated by hydrodistillation from (**a**) *B. daphnoides* leaves, (**b**) *B. daphnoides* stems, (**c**) *B. daphnoides* flowers, (**d**) *B. daphnoides fruits* and (**e**) dehydroepingaione.

**Figure 2:**
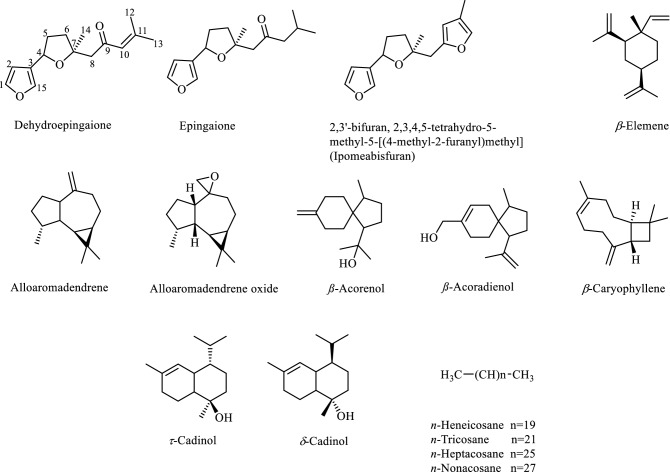
Structures of the major chemical constituents from *B. daphnoides* essential oils.

The volatile constituents of the leaves, stems, flowers and fruits of *B. daphnoides* were qualitatively and quantitatively analyzed using GC/MS. The obtained essential oils of all organs were yellow in color. The identified compounds, their percentages as well as the retention indices are listed in Table 1. GC/MS analysis indicated the presence of 64 components representing 99.37, 97.69, 96.76 and 90.83% of the oils of *B. daphnoides* leaves, stems, flowers and fruits, respectively. They were identified by direct comparison of their mass fragmentation pattern and retention indices with the reported data and computer library search. Quantitative and qualitative results for analysis of these oils are shown in Fig. 1 according to their elution sequences and retention indices^18^. The chemical structures of the major identified constituents are illustrated in Fig. 2.

**Table 1:** Volatile constituents of *B. daphnoides* leaves (BDL), *B. daphnoides* stems (BDS), *B. daphnoides* flowers (BDF) and *B. daphnoides* fruits (BDFR).

No	Retention time	Name	Molecular formula	RI		Relative content %				Identification method
				Calculated	Reported	BDL	BDS	BDF	BDFR	
1	8.28	Benzaldehyde	C_7_H_6_O	950	952	0.15	–	–	–	MS, RI
2	8.40	Methyl 2-methylhexanoate	C_8_H_16_O_2_	967	969	0.05	0.39	–	–	MS, RI
3	8.67	*β*-Pinene	C_10_H_16_	976	976	0.01	0.07	–	–	MS, RI
4	10.16	*p*-Cymene	C_10_H_14_	1016	1016	0.01	0.01	–	–	MS, RI
5	10.28	Limonene	C_10_H_16_	1020	1020	0.3	0.26	–	–	MS, RI
6	11.22	*γ*-Terpinene	C_10_H_16_	1051	1051	0.01	–	–	–	MS, RI
7	11.38	Methyl 2-methylheptanoate	C_9_H_18_O	1066	1067	0.02	0.28	–	–	MS, RI
8	20.00	*α*-Longipinene	C_15_H_24_	1344	1343	0.01	0.05	0.07	–	MS, RI
9	20.57	*α*-Ylangene	C_15_H_24_	1365	1365	0.03	0.04	0.06	–	MS, RI
10	20.70	*α*-Copaene	C_15_H_24_	1369	1369	0.03	0.04	0.11	–	MS, RI
11	21.13	*β*-Elemene	C_15_H_24_	1384	1384	0.53	0.42	1.98	–	MS, RI
12	21.89	*β*-Caryophyllene	C_15_H_24_	1412	1412	0.24	0.27	0.98	–	MS, RI
13	22.72	*α*-Himachalene	C_15_H_24_	1444	1444	0.03	0.06	0.24	–	MS, RI
14	22.83	*α* -Caryophyllene (Humulene)	C_15_H_24_	1449	1449	0.12	0.15	0.47	–	MS, RI
15	23.37	*γ*- Gurjunene	C_15_H_24_	1470	1477	–	–	0.24	–	MS, RI
16	23.49	*β*-Chamigrene	C_15_H_24_	1474	1474	0.02	–	–	–	MS, RI
17	23.62	Alloaromadendrene	C_15_H_24_	1479	1478	10.37	4.32	8.62	0.26	MS, RI
18	23.92	*β*-Guaiene	C_15_H_24_	1491	1491	0.01	0.18	0.43	–	MS, RI
19	24.07	*β*-Himachalene	C_15_H_24_	1497	1497	0.05	0.23	0.81	–	MS, RI
20	24.20	4*β*H,5*α*-Eremophila-1(10),11-diene (Isoeremophilene)	C_15_H_24_	1501	1500	0.05	0.13	0.3	–	MS, RI
21	24.38	Germacrene-D	C_15_H_24_	1509	1510	–	0.08	0.07	–	MS, RI
22	24.61	***δ***-Cadinene (Cadina-1(10),4-diene)	C_15_H_24_	1517	1517	0.13	0.34	0.83	–	MS, RI
23	24.83	Cadina-1,4-diene	C_15_H_24_	1527	1528	–	0.06	–	–	MS, RI
24	25.14	*α*-Calacorene	C_15_H_20_	1539	1539	–	0.03	–	–	MS, RI
25	25.29	Elemol	C_15_H_26_O	1544	1544	0.02	0.13	–	–	MS, RI
26	26.17	Caryophyllene oxide	C_15_H_24_O	1579	1580	–	0.17	0.11	0.44	MS, RI
27	26.72	Humulene epoxide II	C_15_H_24_O	1601	1601	–	–	–	0.27	MS, RI
28	26.89	Cubenol	C_15_H_26_O	1609	1609		0.06	0.35	–	MS, RI
29	27.03	Junenol	C_15_H_26_O	1615	1618	–	0.05	0.18	–	MS, RI
30	27.26	*α*-Acorenol	C_15_H_26_O	1624	1626	0.07	0.31	0.68	–	MS, RI
31	27.59	*δ*-Cadinol	C_15_H_26_O	1627	1622	0.08	0.32	1.03	–	MS, RI
32	27.77	*β*-Acorenol	C_15_H_26_O	1645	1648	0.31	1.33	3.10	–	MS, RI
33	27.90	*τ*-Cadinol	C_15_H_26_O	1651	1651	0.11	–	3.98	–	MS, RI
34	27.95	Alloaromadendrene oxide	C_15_H_24_O	1652	1650	0.19	1.42	–	–	MS, RI
35	28.06	(4aR,5R,9aR)-1,1,4a,8-Tetramethyl-2,3,4,4a,5,6,7,9a-octahydro-1H-benzo[7]annulen-5-ol (Allohimachalol)	C_15_H_26_O	1675	1674	–	0.13	0.52	–	MS, RI
36	28.20	Aromadendrene oxide-(2)	C_15_H_24_O	1680	1678	–	–	–	0.15	MS, RI
37	28.37	8-Isopropyl-1,5-dimethyltricyclo[4.4.0.02,7]dec-4-en-3-one	C_15_H_22_O	1688	1687	–	–	–	0.22	MS, RI
38	28.65	Epingaione	C_15_H_22_O_3_	1699		0.18	7.40	6.28	49.72	MS^32^
39	29.02	2,3’-Bifuran, 2,3,4,5-tetrahydro-5-methyl-5-[(4-methyl-2-furanyl)methyl]	C_15_H_18_O_3_	1713		2.14	5.42	2.09	0.27	MS
40	29.14	6-Isopropenyl-4,8a-dimethyl-1,2,3,5,6,7,8,8a-octahydro-naphthalen-2-ol	C_15_H_24_O	1717	1714	–	–	0.23	0.28	MS, RI
41	29.29	2,6-Nonadien-4-one, 9-(3-furanyl)-2,6-dimethyl-, (*E*)	C_15_H_20_O_2_	1726		0.15	0.1	0.16	–	MS
42	29.70	(*Z*)-*α*-Bisabolene epoxide	C_15_H_24_O	1730	1733	–	0.3	–	–	MS, RI
43	30.40	Dehydroepingaione	C_15_H_20_O_3_	1760		83.60	72.36	58.78	34.18	MS
44	30.80	*β*-Acoradienol	C_15_H_24_O	1778	1768	–	–	–	1.58	MS, RI
45	31.76	2,5-Nonadien-4-one, 9-(3-furanyl)-2,6-dimethyl-, (E) (trans-Dihydrophymaspermone)	C_15_H_20_O_2_	1823		0.07	0.1	–	–	MS
46	32.44	2,5,7-Nonatrien-4-one, 9-(3-furanyl)-2,6-dimethyl-, (*E,E*) (Phymaspermone)	C_15_H_18_O_2_	1855		0.04	0.05	–	–	MS
47	33.01	Nonadecane	C_19_H_40_	1899	1900	–	–	0.12	–	MS, RI
48	33.03	9,12,15-Octadecatrien-1-ol, (*Z,Z,Z*)-	C_18_H_32_O	1901		0.06	–	–	–	MS
49	34.39	*n*-Hexadecanoic acid	C_16_H_32_O_2_	1968	1968	0.04	–	–	–	MS, RI
50	35.02	*n*-Eicosane	C_20_H_42_	1999	2000	–	–	0.16	–	MS, RI
51	36.23	Kaur-16-ene	C_20_H_32_	2061	2061	–	–	0.18	–	MS, RI
52	36.94	9,12-Octadecadienoic acid, methyl ester	C_19_H_34_O_2_	2098	2101	–	–	–	0.16	MS, RI
53	36.94	*n*-Heneicosane	C_21_H_44_	2098	2100	–	–	2.06	–	MS, RI
54	37.89	Linolenic acid	C_18_H_30_O_2_	2149	2147	0.04	–	–	–	MS, RI
55	38.81	*n*-Docosane	C_22_H_46_	2199	2200	–	–	0.13	–	MS, RI
56	40.58	*n*-Tricosane	C_23_H_48_	2298	2300	–	0.03	1.32	–	MS, RI
57	43.93	*n*-Pentacosane	C_25_H_52_	2499	2500	0.02	0.04	0.09	0.20	MS, RI
58	45.51	*n*-Hexacosane	C_26_H_54_	2598	2600	–	0.01	–	–	MS, RI
59	47.02	*n*-Heptacosane	C_27_H_56_	2697	2700	0.05	0.3	–	1.11	MS, RI
60	48.11	3-Methylheptacosane	C_28_H_58_	2771	2771	–	0.01	–		MS, RI
61	48.47	*n*-Octacosane	C_28_H_58_	2797	2800	–	0.01	–	0.14	MS, RI
62	49.89	*n*-Nonacosane	C_29_H_60_	2896	2900	0.03	0.18	–	1.42	MS, RI
63	50.93	3-Methylnonacosane	C_30_H_62_	2970	2970	–	0.01	–	0.14	MS, RI
64	52.59	*n*-Untriacontane	C_30_H_62_	3095	3100	–	0.04	–	0.29	MS, RI
Monoterpenes						0.33	0.34	0	0	
Sesquiterpenes						11.62	6.4	15.21	0.26	
Oxygenated sesquiterpenes						86.96	89.65	77.49	87.11	
Others						0.46	1.3	4.06	3.46	
Total identified components						99.37	97.69	96.76	90.83	

The names of the components are in order of their elution from the Rtx-5MS column.

Identification, was based on comparison of the compounds’ mass spectral data (MS) and retention indices (RI) with those of NIST Mass Spectral Library and Adams.

Analysis of the volatile constituents of *B. daphnoides* leaves revealed the presence of 38 compounds. Dehydroepingaione (83.6%), an oxygenated sesquiterpene, represented the major constituent followed by alloaromadendrene (10.37%) and 2,3’-bifuran, 2,3,4,5-tetrahydro-5-methyl-5-[(4-methyl-2-furanyl)methyl] (2.14%). From the stems’ oil, 45 components were identified where dehydroepingaione was the major constituent (72.36%) followed by epingaione (7.4%), 2,3’-bifuran, 2,3,4,5-tetrahydro-5-methyl-5-[(4-methyl-2-furanyl)methyl] (5.42%) and alloaromadendrene (4.32%). Thirty four compounds were identified from the oils of flowers comprising mainly dehydroepingaione (58.78%), alloaromadendrene (8.62%) and epingaione (6.28%). From the fruits’ oil, 17 components were identified including epingaione as a predominant constituent (49.72%), followed by dehydroepingaione (34.18%).

### Spectroscopic data of dehydroepingaione

The compound was isolated for the first time from the *n*-hexane fraction of *B. daphnoides* leaves as a yellow oil; with R_*f*_ = 0.44 in *n*-hexane: EtOAc (9:1). ^1^H NMR (400 MHz, CDCl_3_), ^13^C NMR (100 MHz, CDCl_3_) and 2D NMR spectroscopic data are displayed in Supplementary Table S1 and Fig. S2. The NMR data were in agreement with the corresponding data described in literature^14,17,19^.

### Quantitative analysis of dehydroepingaione using standard calibration curve

A linear calibration curve of different concentration of dehydroepingaione vs. peak area was obtained and the peak area of 1 mg/mL of the essential oils of *B. daphnoides* leaves, stems, flowers and fruits were 50,431,735, 39,300,212, 36,915,263 and 22,546,502, respectively. The concentration of dehydroepingaione was calculated from the standard curve displayed in Fig. S3, where it was 890.09, 695.99, 654.41 and 403.87 µg/mg in the oil of the leaves, stems, flowers and fruits, respectively.

### Multivariate analysis for discrimination of different organs of B. daphnoides

**Figure 3:**
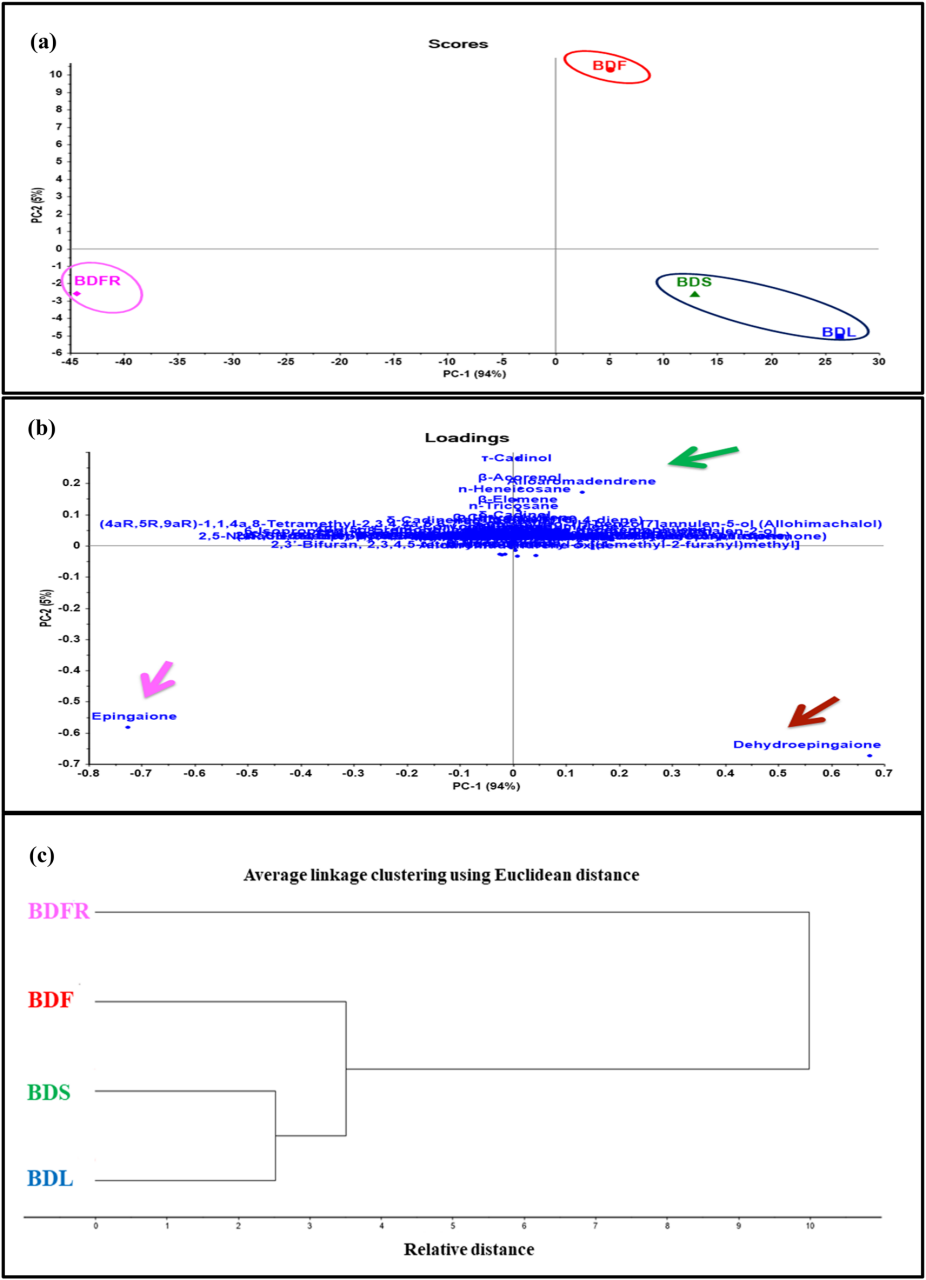
GC based chemometrics analysis of different organs of *B. daphnoides* (**a**) score plot; (**b**) loading plot; (**c**) HCA.

Multivariate data analysis was performed utilizing unsupervised pattern recognition techniques; PCA and HCA, relying upon the results of the average data of three GC/MS runs to differentiate the different organs of *B. daphnoides*. Figure 3a showed the score plot which demonstrated the efficient discrimination of the different organs into three distant clusters where the leaves and stems were distributed in the same cluster in contrast to the flowers and fruits that appeared in separate clusters. PCA score plot for principal components (PCs), PC1 versus PC2, accounted for 94 and 5% of the total variance, respectively. The fruits and flowers could be completely discriminated along both PC1 and PC2 where the cluster of the flowers lied in the upper right quadrant displaying positive values for PC1 and PC2 in contrast to the cluster of the fruits that was located in the lower left quadrant showing negative values for PC1 and PC2. At the same time, PC1 efficiently differentiated between the fruits with negative values and leaves along with stems that lied in the lower right quadrant with positive values. Additionally, PC2 differentiated between flowers that showed positive PC2 values and leaves, stems, and fruits that showed negative PC2 values. Comprehensive interpretation of the loading plot (Fig. 3b) showed that epingaione, dehydroepingaione, 2,3’-bifuran, 2,3,4,5-tetrahydro-5-methyl-5-[(4-methyl-2-furanyl)methyl], *n*-heneicosane, alloaromadendrene, *β*-acorenol, *τ*-cadinol, *β*-elemene, and *n*-tricosane represented the predominant discriminatory components in the hydrodistillation products of the four organs. Moreover, HCA was performed with the clustering dendrogram obtained from HCA showed the differentiation of organs into three discriminative clusters (displayed in Fig. 3c). Both leaves and stems were clustered together compared to fruits and flowers that formed two clusters.

### In vitro antiviral activity

Antiviral activity was assessed against HSV-1 and CoxB4 using MTT assay in Vero cell. Samples were tested at the maximum non-toxic concentration (MNTC) of each sample on Vero cells which was 3.125 µg/ml for the essential oils of the leaves and stems, 12.5 µg/ml for the flowers and dehydroepingaione, while it was 1.56 µg/ml for the fruits’ oil. The essential oil isolated from the fruits showed the highest toxicity on Vero cells (CC_50_ = 1.64 µg/ml) and the highest inhibition of viral activity 61.75 and 58.53% against HSV-1 and CoxB4. The leaves and stems of *B. daphnoides* showed similar activity with IC_50_ values ranging from 11.98 to12.62 µg/ml against HSV-1 and IC_50_ values of 13.34–14.5 µg/ml against CoxB4. The oil of the flowers showed the least activity with IC_50_ of 25.60 and 31.12 µg/ml against HSV-1 and CoxB4, respectively. The antiviral activity was also measured for the isolated compound dehydroepingaione which showed promising activity against HSV-1 with IC_50_ = 24.46 µg/ml and against CoxB4 with IC_50_ = 25.32 µg/ml. The selectivity index (SI) was calculated for the oils of the leaves, stems and flowers with dehydroepingaione where they showed SI values ranging from 1.08 to 1.45. The results are displayed in Table 2 and Supplementary Fig. S4.

**Table 2:** Cytotoxicity and antiviral activity of the essential oils of the leaves, stems, flowers and fruits of *B. daphnoides* and the isolated compound (dehydroepingaione).

Sample	CC_50_^a^ (µg/ml) Vero cells	Inhibition % HSV-1	Inhibition % CoxB4	IC_50_ (µg/ml) ± SD^c^ HSV-1	SI	IC_50_^b^ (µg/ml) ± SD^c^ CoxB4	SI
BDL	17.34 ± 0.89	33.18	21.36	11.98 ± 1.6	1.45	13.34 ± 1.6	1.29
BDS	18.11 ± 0.74	37.57	17.17	12.62 ± 2.3	1.43	14.52 ± 2.3	1.24
BDF	36.33 ± 0.39	43.64	25.46	25.60 ± 0.99	1.41	31.12 ± 0.99	1.16
BDFR	1.64 ± 0.31	61.75	58.53	NA^e^	NA^e^	NA^e^	NA^e^
Dehydroepingaione	27.54 ± 0.32	43.86	41.07	24.46 ± 2.3	1.12	25.32 ± 1.7	1.08
Acyclovir	7.1 ± 0.22			0.64 ± 0.34	11.09	NA^e^	NA^e^

^a^Toxic concentration to 50% of the Vero cells.

^b^Concentration required to inhibit 50% of the viral growth .

^c^Standard deviation.

^d^SI, selectivity index (= CC_50_/IC_50_).

^e^Not applicable.

### In silico molecular modelling

In silico virtual screening was performed using the major identified volatile components from the essential oils of different organs of *B. daphnoides* on two enzymes including, HSV type-1 thymidine kinase (TK) and HSV type-1 DNA polymerase (DP). Results illustrated in Table 3 revealed that *n*-heneicosane and 2,3’-bifuran, 2,3,4,5-tetrahydro-5-methyl-5-[(4-methyl-2-furanyl)methyl] showed inhibition to both enzymes where *n*-heneicosane exhibited the best fitting within the active site of TK displaying binding energy (∆G) of −25.88 kcal/mol, meanwhile it showed (∆G of −43.12 kcal/mol for DP exceeding the value of the standard drug acyclovir. Furthermore, 2,3’-bifuran, 2,3,4,5-tetrahydro-5-methyl-5-[(4-methyl-2-furanyl) methyl] showed (∆G of −19.77 and −15.63 kcal/mol, for TK and DP, respectively. Additionally, dehydroepingaione and epingaione showed similar inhibition of TK with ∆G of −12.66 and −11.73 kcal/mol, respectively. Long chain alkanes as *n*-heptacosane, *n*-nonacosane and *n*-tricosane showed good fitting to DP with ∆G of −49.15, −52.22, and −44.21 kcal/mol, respectively.

**Table 3:** Binding energies (kcal/mol) of the major identified compounds from different organs of *B. daphnoides* in HSV type-1 thymidine kinase and HSV type-1 DNA polymerase active sites using molecular modelling experiment.

Compounds	HSV type-1 thymidine kinase	Number of formed Hydrogen bonds	Number of formed Alkyl and π-Alkyl Bonds	HSV type-1 DNA polymerase	Number of formed Hydrogen bonds	Number of formed Alkyl and π-Alkyl Bonds
Dehydroepingaione	**−12.66**	2; Arg163;Arg176	9; Ala168, Arg222, His58, Ile97, Ile100, Met128, Trp88, Tyr132, Tyr172	0.94	1; Lys539	4; Phe381, Phe470, Pro382, Tyr557
Epingaione	**−11.73**	1; Arg176	10; Ala167, Ala168, Arg222, His58, Ile97, Ile100, Met128, Trp88, Tyr132, Tyr172	−0.35	2; Arg75, Lys484	1; Ile482, Pro387
2,3’-Bifuran, 2,3,4,5-tetrahydro-5-methyl-5-[(4-methyl-2-furanyl)methyl]	−**19.77**	1; Arg163	5; Ala168, His58, Ile97, Met128, Tyr101	−**15.63**	–	–
*n*-Heneicosane	−**25.88**	–	–	−**43.12**	–	–
*n-*Heptacosane	FD	–	–	**−49.15**	–	–
*n-*Nonacosane	FD	–	–	**−52.22**	–	3; Lys539, Tyr465, Tyr526
*n-*Tricosane	FD	–		**−44.21**	–	–
Alloaromadendrene oxide	15.27	–	9; Arg222, His58, Ile97, Ile100, Met128, Met231, Trp88, Tyr101, Tyr172	20.68	1; Lys539	2; Leu540, Phe470
Alloaromadendrene	19.79	–	6; His58, Ile97, Ile100, Met128, Trp88, Tyr172	27.03	–	1; Phe470
*δ*-Cadinol	8.44	1; Glu83	7; His58, Ile97, Ile100, Met128, Trp88, Tyr101, Tyr172	15.17	1; Asp368	2; Phe381, Phe470
*β*-Acoradienol	34.63	1; Arg176	5; Arg222, His58, Ile97, Trp88, Tyr172	37.53	1; Asp554	–
*β*-Acorenol	31.70	1; Tyr132	8; Arg222, His58, Ile97, Ile100, Met231, Trp88, Tyr101, Tyr172	27.14	1; Glu370	1; Phe470
*β*-Elemene	26.06	–	7; His58, Ile97, Ile100, Met128, Trp88, Tyr101, Tyr172	29.03	–	3; Phe381, Phe470, Pro382
*τ*-Cadinol	15.04	–	6; Arg222, His58, Ile97,Met128, Trp88, Tyr172	16.45	1; Lys539	1; Phe470
Acyclovir	**−35.12**	5; Arg163, Arg176, Arg222, Lys62, Tyr 101	1; Arg222	**−32.90**	5; Asp368, Cys371, Glu370, Leu540, Lys539	1; Leu540

Significant values are bold.

**Figure 4:**
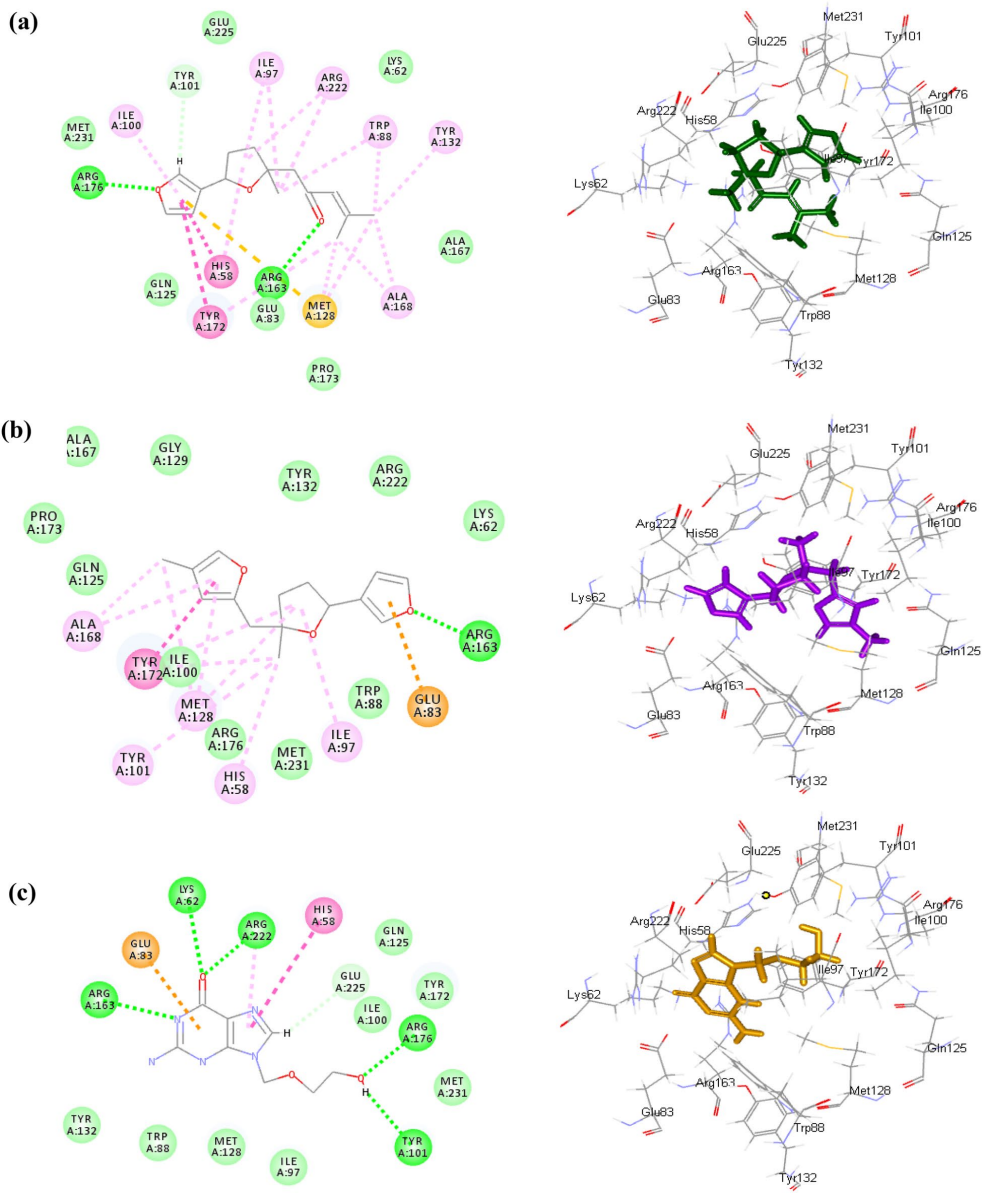
2D and 3D interactions of dehydroepingaione (**a**), 2,3’-bifuran, 2,3,4,5-tetrahydro-5-methyl-5-[(4-methyl-2-furanyl)methyl] (**b**) and acyclovir (**c**) in the active site of HSV type-1 thymidine kinase (TK).

**Figure 5:**
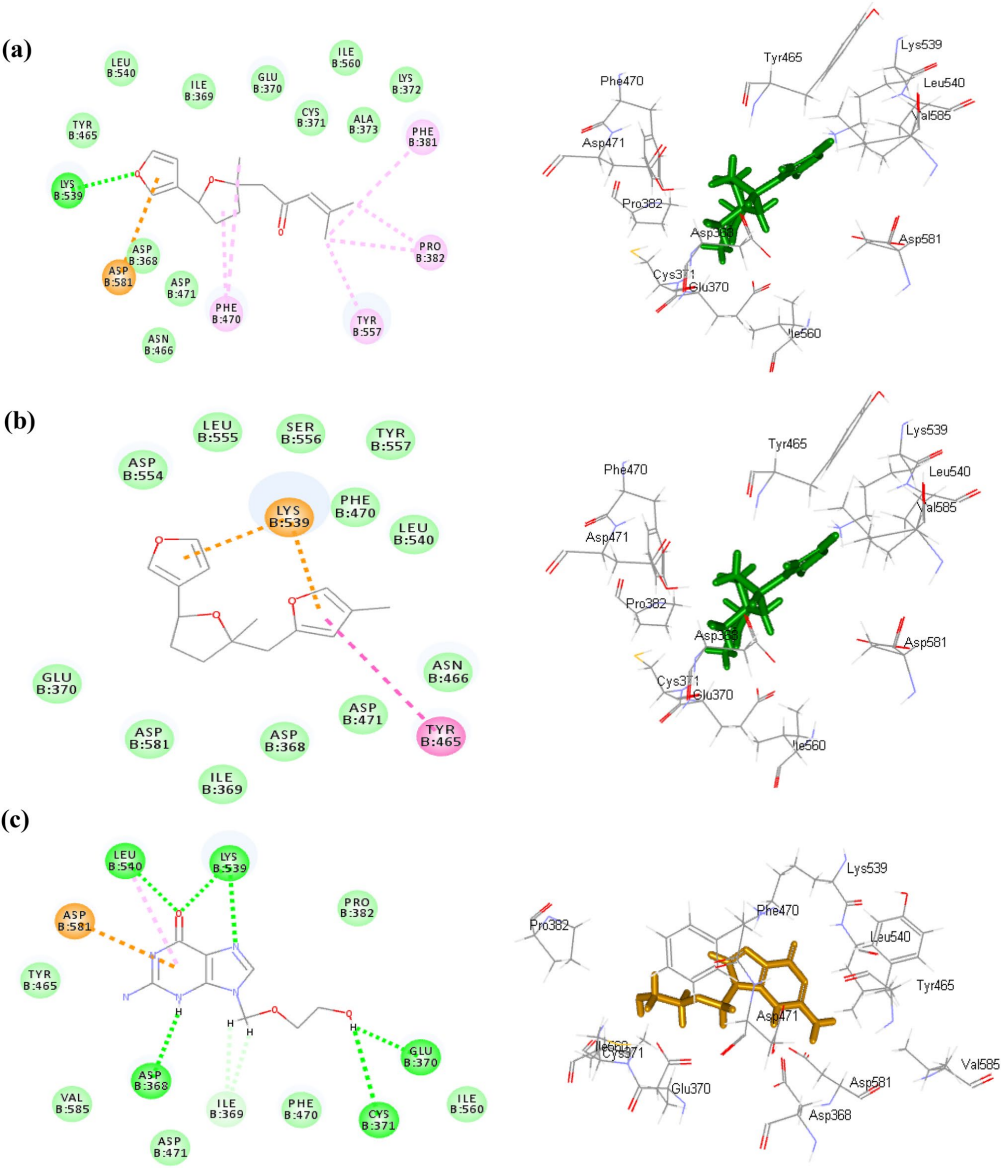
2D and 3D interactions of dehydroepingaione (**a**), 2,3’-bifuran, 2,3,4,5-tetrahydro-5-methyl-5-[(4-methyl-2-furanyl)methyl] (**b**) and acyclovir (**c**) in the active site of HSV type-1 DNA polymerase (DP).

This firm fitting of the compounds within the active site of the enzymes can be explained by their favorable binding through the formation of several bonds. Concerning TK, *n*-heneicosane formed one π-sigma bond with Tyr172 at the active site along with many Van der Waals interactions. While 2,3′-bifuran, 2,3,4,5-tetrahydro-5-methyl-5-[(4-methyl-2-furanyl)methyl] formed one conventional H- bond with Arg163, one π-anion bond with Glu83, one π- π bond with Tyr172 and five alkyl/ π-alkyl bonds with Ala168, His58, Ile97, Met128 and Tyr101 in addition to Van der Waals interactions. Dehydroepingaione and epingaione showed similar interactions within the active site of TK where both formed conventional H- bonds with Arg176, in addition to the formation of many alkyl/π-alkyl bonds and Van der Waals interactions (Fig. 4). Regarding DP, *n*-heneicosane formed many Van der Waals interactions within the active site while 2,3’-bifuran, 2,3,4,5-tetrahydro-5-methyl-5-[(4-methyl-2-furanyl)methyl] formed one π- π bond with Tyr465, one π-cation bond with Lys539 in addition to multiple Van der Waals interactions. Long chain alkanes as *n*-heptacosane, *n*-nonacosane and *n*-tricosane formed many Van der Waals interactions within the active site of DP. Meanwhile *n*-nonacosane formed three alkyl/ π-alkyl bonds with Lys539, Tyr465, Tyr526 (Fig. 5). Acyclovir was used as the reference antiviral drug and showed the formation of five conventional H- bond within the active sites of both DP and TK together with one π-alkyl bond, one π-anion and many Van der Waals interactions (Figs. 4 and 5).

## Discussion

Using GC/MS techniques, 64 components were identified from the essential oils of *B. daphnoides* leaves, stems, flowers and fruit. The oil yields were 0.35, 0.11, 0.08 and 0.25% w/w for the leaves, stems, flowers and fruits, respectively. The leaves showed the highest yield and the flowers showed the lowest yield among the different organs. The data displayed in Table 1 indicated that oxygenated sesquiterpenes were predominant as the main class of volatile constituents where they represented 86.96, 89.65, 77.49 and 87.11% of the oil content in leaves, stems, flowers and fruits, respectively. Dehydroepingaione, an oxygenated sesquiterpene, represented 83.60, 72.36, 58.78 and 34.18% of the oils of the leaves, stems, flowers and fruits, respectively. Epingaione which was isolated previously from the leaves extract represented 6.28, 7.4 and 49.72% of the volatile constituents of the flowers, stems and fruits^14,17^. Alloaromadendrene sesquiterpene represented 10.37, 8.62, 4.32 and 0.26% in the leaves, flowers, stems and fruits, respectively. Other constituents include sesquiterpenes, monoterpenes, long chain aliphatic alkanes and fatty acids.

Quantitative analysis of dehydroepingaione in the essential oils of different organs of *B. daphnoides* was performed using standard calibration curve. The leaves showed the highest content followed by the stems, the flowers and the fruits which showed the lowest content. These results matched the results of GC/MS quantitative analysis of dehydroepingaione by relative peak area %.

Chemometric analysis of different organs of *B. daphnoides* was done based on the qualitative and quantitative GC data utilizing PCA and HCA which showed the differentiation of organs into three discriminative clusters. Both leaves and stems were clustered together compared to fruits and flowers that formed two clusters. These results indicated the similarity in the secondary metabolites of the leaves and stems that may affect their bioactivities compared to fruits and flowers^20^.

The cytotoxicity of the essential oils and isolated compound was measured on Vero cell lines and the antiviral activity was detected on HSV-1 and CoxB4 viruses at the MNTC. The essential oil of the fruits exhibited the highest inhibition of viral growth and the lowest CC_50_ on Vero cells among the tested oils. It is recommended that this oil should be reassessed for its safe and effective dose as antiviral agent. Meanwhile, the essential oils from the leaves, stems and flowers showed antiviral activity at the MNTC on Vero cells where the leaves’ and stems’ oils showed better activity than flowers’ oil. Dehydroepingaione, the major sesquiterpenes, exhibited antiviral activity against HSV-1 and CoxB4.

The oil samples need re-evaluation using other bioassays for confirmation of their safety as the calculated SI value is between 1 and 10^21^. The results of in vitro antiviral activity further confirmed the similarity between the oils of the leaves and stems and their differentiation from the fruits and flowers.

Molecular modelling study of the major constituents from different organs of *B. daphnoides* highlighted the ability of the identified constituents to inhibit critical enzymes such as HSV type-1 TK and HSV type-1 DP that are commonly used as molecular targets for screening of the activity against HSV-1^1,22–24^. The reference antiviral drug (Acyclovir) was reported to have high affinity to TK causing its phosphorylation and incorporation in viral DNA which lead to binding to DP, blocking DNA synthesis of the virus and leading to antiviral activity^25^. The results of this study showed the affinity of sesquiterpenes as dehydroepingaione (the major component of the leaf, stem and flower oils) and epingaione (the major component of the fruit oil) towards TK due to the formation of multiple tight hydrogen bonds with the amino acid moieties within the active sites. Other components as 2,3’-bifuran, 2,3,4,5-tetrahydro-5-methyl-5-[(4-methyl-2-furanyl)methyl] and *n*-heneicosane showed good affinity towards both enzymes. Moreover, long chain alkanes as *n*-heptacosane, *n*-nonacosane and *n* tricosane showed strong affinity towards DP.

These results further ascertain the obtained in vitro results where the fruits’ oil showed the highest inhibition of HSV-1 due to high content of oxygenated sesquiterpenes as epingaione and dehydroepingaione together with *n*- long chain alkanes as *n*-heptacosane and *n*-nonacosane. While the oils of the leaves and stems showed similar activity which may be attributed to the major oxygenated sesquiterpenes as dehydroepingaione, 2,3’-bifuran, 2,3,4,5-tetrahydro-5-methyl-5-[(4-methyl-2-furanyl)methyl] and epingaione. The flowers showed lower activity where active oxygenated sesquiterpenes exist in lower percentages. However other sesquiterpenes as alloaromadendrene, alloaromadendreneoxide, *δ*-cadinol, *β*-acoradienol, *β*-acorenol, *β*-elemene, and *τ*-cadinol showed weak fitting to both enzymes. This ensures that the activity might be attributed to the major oxygenated sesquiterpenes; dehydroepingaione and epingaione together with 2,3’-bifuran, 2,3,4,5-tetrahydro-5-methyl-5-[(4-methyl-2-furanyl)methyl] which showed good antiviral activity by targeting TK, however these compounds showed low affinity on DP as revealed by molecular docking studies.

The obtained results were in agreement with many studies which proved the antiviral activities of medicinal plants belonging to various genera in tribe Myoporeae as *Myoporum* and *Eremophila*. The chemical characterization and biological activities of different members from *Myoporum* species showed its richness in essential oils that compose mainly of sesquiterpenes and oxygenated sesquiterpenes with insecticidal, antibacterial, antifungal and antiviral activities^19,26–28^. Furthermore the essential oil from different species of *Eremophila* genus showed promising antibacterial, antifungal and antiviral activities^29,30^. Epingaione and dehydroepingaione were isolated from *M. bontioides* and exhibited anti-MRSA effect^19^. Also, epingaione isolated previously from the leaves of *B. daphnoides* showed insecticidal activity with LC_50_ 20.80 (μg/insect)^17^. Therefore, the antiviral activity of essential oils from different organs of *B. daphnoides* is attributed to their content of oxygenated sesquiterpenes mainly dehydroepingaione, epingaione and 2,3’-bifuran, 2,3,4,5-tetrahydro-5-methyl-5-[(4-methyl-2-furanyl)methyl]. As some furanoid sesquiterpenes may have certain toxicity and the calculated selectivity index is low, the oils of *B. daphnoides* must be reconsidered for the effective concentration and toxic dose before using as antiviral agents. In addition to safety concerns, this study may have potential limitations due to the scarcity of data on the genus as it is monotypic genus that includes only one species (*B. daphnoides*). This point can be considered as an opportunity to fill in literature gaps and to present the necessity for more studies on this species. Also the limited access of proteins targets for testing the in silico activity against CoxB4 virus may be considered as another limitation to this study.

## Conclusion

In this study, GC/MS of the essential oils from the leaves, stems, flowers and fruits of *B. daphnoides* showed the variation in the volatile constituents among the different organs. Oxygenated sesquiterpenes were the major class of volatile constituents. Chemometric study was performed based on GC data where PCA and HCA revealed the similarity of the leaves and stems as they were located in the same cluster while the fruits and flowers were distributed in separate clusters. The major compound (dehydroepingaion) was isolated from the lipophilic fraction of the leaves extract and its concentration in the different organs was determined using standard calibration curve. The leaves’ and stems’ oils showed good antiviral activity against HSV-1 and CoxB4 while the flowers oil showed lower activity. Moreover, dehydroepingaione displayed activity against the tested viruses. In silico molecular modeling gave a prediction of molecular interactions’ mechanism where the antiviral activity of the oils was attributed to the major identified oxygenated sesquiterpenes. Consequently, the ongoing study reveals that the oils of *B. daphnoides* have antiviral activity which is matching with the folk medicinal uses of the plant. Using the oils as natural antiviral drug needs more in depth studies to confirm their activity and safety. Furthermore, extra studies are recommended toward the antiviral activity of the oils and identified compounds against the pandemic coronavirus disease 2019 (COVID-19).

## Materials and methods

### Plant material

The fresh leaves, stems, flowers and fruits of *B. daphnoides* L. were collected from EgyGerman Agricultural Company, Sharkeya, Egypt (30°47′43.0ʺN 32°03′52.4ʺE), in July, 2018. The collection of plant material had received permission from the company owner and complied with the relevant institutional, national, and international guidelines and legislation. During the collection, we took all care not to cause any damage to the species. The plant was identified and authenticated morphologically by Eng. Therease Labib, consultant of plant taxonomy at the Ministry of Agriculture, National Gene Bank and El-Orman Botanical Garden, Egypt. Plant specimen with code PHG-P-BD-318 was deposited as a voucher specimen at the herbarium of Pharmacognosy Department, Faculty of Pharmacy, Ain Shams University, Cairo, Egypt (Supplementary Fig. S1). Also a sample of the plant has been transported to the Medicinal Plants Research Station at Faculty of Pharmacy, Ain Shams University, Cairo, Egypt, and it was successfully cultivated.

### Materials for phytochemical investigation

Shimadzu GC/MS QP-2010 (Shimadzu Corporation, Kyoto, Japan) was used to record GC mass spectrum. Silica gel (Kieselgel 60 A°, 70–230 mesh, 63–200 µ, Fluka, Sigma Aldrich, Germany) was used for column chromatography. TLC analysis and preparative TLC were performed utilizing precoated normal phase silica gel plates F254 (Merck, Germany). Vanillin/ H_2_SO_4_ reagent was used for spraying and visualizing TLC spots followed by heating on a hot plate at 100 °C. Solvents used for extraction and fractionation were of high purity (distilled). ^1^H, ^13^C (APT) and 2D NMR analyses were performed using Bruker Ascend 400/R spectrometer (Burker Avance III, Fallanden Switzerland) at the Center for Drug Discovery, Research and Development, Faculty of Pharmacy, Ain Shams University using the operating frequencies of 400 and 100 MHz, respectively. The sample was dissolved in deuterated chloroform (Sigma Aldrich, Germany) and transferred to 3 mm NMR tubes (Bruker). Tetramethylsilane (Me_4_Si) was used as the internal standard.

### Materials for biological study

Vero cells (ATCC No. CCL-81) isolated from kidney epithelial cells and extracted from African green monkey were obtained commercially. Rapidly growing virus strains producing a cytopathic effect in Vero cells within three days were used as HSV-1 and CoxB4. Other chemicals and reagents as 3-[4-dimethylthiazol-2-yl]-2,5-diphenyl tetrazolium bromide solution (MTT) (BIO BASIC CANADA INC), acyclovir (9-(2-hydroxyetho-xymethyl)guanosine, Sigma), growth medium and DMSO were purchased at the highest possible purity.

### Isolation of *B. daphnoides* essential oils

The fresh leaves, stems, flowers and fruits of *B. daphnoides*, (150 g for leaves, stems and fruits and 50 g for flowers) were subjected to hydrodistillation using a Clevenger-type apparatus for four hours. Extracted oils were kept for further analyses in separate, sealed vials at 4 °C. The yield was determined and expressed in % *w/w* based on the initial plant weight.

### Gas chromatography/mass spectrometry (GC/MS) analysis

GC/MS analysis was performed using Shimadzu GC/MS QP-2010 equipped with Rtx-5MS fused bonded column (30 m × 0.25 mm i.d. × 0.25 µm film thickness) (Restek, USA). The used conditions were as previously described^31^. Volatile components were identified by direct comparison of their retention indices (RI) and mass spectral data with NIST Mass Spectral Library and literature^18,32–36^. The relative content of each peak was calculated based on the percentage of peak area relative to the total peak area. RI was calculated relative to a homologous series of *n*-alkanes (C8-C28) injected under the same conditions^37^.

### Isolation of dehydroepingaione

The crushed air-dried leaves of *B. daphnoides* (3.2 kg) were percolated in distilled methanol (12 L × 3) then filtered. The filtrate was completely evaporated in vacuum at low temperature (45 °C) till dryness to yield 622 g. The dried extract (592 g) was then fractionated with *n*-hexane, dichloromethane, ethyl acetate and *n*-butanol, successively to give 122.9 g, 92.34 g, 54.47 g and 61 g, respectively. The *n*-hexane fraction (45 g) was further subjected to fractionation with 70% methanol to give 70% methanol and *n*-hexane fractions. 70% methanol fraction (17 g) was chromatographed on 280 g silica gel using the dry loading method. The column was eluted using mixtures of *n*-hexane–ethyl acetate with increasing polarity as eluents till 100% methanol. Similar fractions were pooled together to give 19 major fractions. Fraction V (100 mg) was eluted with a mixture of *n*-hexane: EtOAc (9:1) and purified over preparative TLC using the same solvent system which resulted in the separation of dehydroepingaione (13 mg) as oily material that showed strong quenching under short UV light and violet color after spraying with vanillin/ H_2_SO_4_ and heating on a hot plate at 100 °C. NMR analysis was carried out for compound identification, also the compound was subjected to GC/MS analysis using the same conditions previously mentioned^37^.

### Quantitative analysis of dehydroepingaione using standard calibration curve

Five different concentrations of dehydroepingaione were prepared (100, 200, 500, 800, 1000 µg/mL) using *n*-hexane as the solvent and subjected to GC/MS analysis. A calibration curve was constructed by plotting the peak area versus the corresponding concentration (µg/mL). Subsequently, essential oils of different organs of *B. daphnoides* were prepared at a concentration of 1 mg/mL and analyzed using GC/MS under the same conditions. Peak area of dehydroepingaione was determined and the concentration was calculated from the standard curve equation y = 57351x–615,723, (R^2^ = 0.9997).

### Multivariate analysis for discrimination of different organs of *B. daphnoides*

The collected GC data were used to carry out chemometric analysis for discrimination of different organs of *B. daphnoides* using unsupervised pattern recognition technique as PCA and HCA. PCA classified the samples into discriminant classes regarding the quality and quantity of the major compounds. Additionally, HCA classified the samples into clusters utilizing the entire linkage approach for group classification. PCA and HCA were performed using CAMO’s Unscrambler® X 10.4 software (Computer-Aided Modeling, As, Norway) as previously described^38^.

### In vitro assessment of cytotoxicity and antiviral activity

#### Determination of samples cytotoxicity on vero cells

A sheet of Vero cell was formed in 96 well micro titer plates then double-fold dilutions of prepared concentrations of tested samples were made and 0.1 ml of each dilution was tested in different wells. Twenty µl of MTT were added to each well and then incubated (37 °C, 5% CO_2_) for 1–5 h. Formed formazan crystals were suspended in 200 µl DMSO then optical density was measured at 560 nm. The toxic concentration to 50% of the Vero cells (CC_50_) was estimated from the concentration-effect curves after linear regression analysis^1^.

#### Determination of antiviral activity via MTT assay protocol

Infection of Vero cells by HSV-1 and CoxB4 was evaluated by quantal assay to have 50% tissue culture infectious dose end-point (TCID50%) and plaque formation unit (PFU). The antiviral activity was evaluated at the MNTC of the tested materials then diluted at different concentrations against TCID50/mL of virus via MTT assay. The viability of infected and non-infected cells was determined using the absorbance of formazan in MTT assay. The viral inhibition percentage was presented as mean of three different experiments values ± SE, t-test (*p* < 0.05) was carried out as appropriate. The IC_50_ is the concentration required to inhibit 50% of the viral growth. Selectivity index was calculated using the equation; (CC_50_/IC_50_). The results were compared to the antiviral drug acyclovir^1^.

### In silico molecular modelling

Molecular modelling was made for the major compounds identified from the essential oils of different organs of *B. daphnoides* within the active sites of HSV type-1 thymidine kinase (PDB ID: 2KI5; 1.90 A°) and HSV type-1 DNA polymerase (PDB ID: 2GV9; 2.68 A°). The structures of the enzymes were downloaded from the protein data bank (PDB). Discovery Studio 4.5 (Accelrys Inc., San Diego, CA, USA) employing C-Docker protocol was used to perform the docking study as previously reported^38,39^. The binding energies (∆ G) for the best docking poses were computed using the previously reported equation^38,40^.

## Supplementary Information

Supplementary Information.

## Data Availability

All data generated during this study are included in this published article and its Supplementary Information file.

## References

[1] Fahmy NM (2020). Breaking down the barriers to a natural antiviral agent: Antiviral activity and molecular docking of *Erythrina speciosa* extract, fractions, and the major compound. Chem. Biodivers..

[2] El-Shiekh RA, Abdelmohsen UR, Ashour HM, Ashour RM (2020). Novel antiviral and antibacterial activities of *Hibiscus schizopetalus*. Antibiotics..

[3] Llitjos JF (2020). High incidence of venous thromboembolic events in anticoagulated severe COVID-19 patients. J. Thromb. Haemost..

[4] Elkousy RH, Said ZN, Abd El-Baseer MA (2021). Antiviral activity of castor oil plant (*Ricinus communis*) leaf extracts. J. Ethnopharmacol..

[5] Shih SR (2004). Selective human enterovirus and rhinovirus inhibitors: An overview of capsid-binding and protease-inhibiting molecules. Med. Res. Rev..

[6] Youssef FS, Altyar AE, Omar AM, Ashour ML (2021). Phytoconstituents, in vitro anti-infective activity of *Buddleja indica* Lam., and in silico evaluation of its SARS-CoV-2 inhibitory potential. Front. Pharmacol..

[7] WFO. World Flora Online. Preprint at http://www.worldfloraonline.org (2022).

[8] Chase MW, Christenhusz MJM, Fay MF, Byng JW, Judd WS, Soltis DE, Mabberley DJ, Sennikov AN, Soltis PS, Stevens PF (2016). An update of the angiosperm phylogeny group classification for the orders and families of flowering plants: APG IV. Bot. J. Linn. Soc..

[9] Thabet AA (2022). Phytochemistry, structural diversity, biological activities and pharmacokinetics of iridoids isolated from various genera of the family Scrophulariaceae Juss. Phytomed. Plus..

[10] Tank DC, Beardsley PM, Kelchner SA, Olmstead RG (2006). Review of the systematics of Scrophulariaceae sl and their current disposition. Aust. Syst. Bot..

[11] Fay MF (2016). The tribe Scrophularieae (Scrophulariaceae): A review of phylogenetic studies. Bot. J. Linn. Soc..

[12] Weng J-R (2017). A flavone constituent from *Myoporum bontioides* induces M-phase cell cycle arrest of MCF-7 breast cancer cells. Molecules.

[13] Chinnock RJ, Ghisalberti EL, Jefferies PR (1987). (-)-Epingaione from *Bontia daphnoides*. Phytochemistry.

[14] Williams LAD (2007). *In vitro* anti-proliferation/cytotoxic activity of epingaione and its derivatives on the human SH-SY5Y neuroblastoma and TE-671 sarcoma cells. West Indian Med. J..

[15] Lans CA (2006). Ethnomedicines used in Trinidad and Tobago for urinary problems and diabetes mellitus. J. Ethnobiol. Ethnomed..

[16] Quattrocchi, U., *CRC world dictionary of medicinal and poisonous plants: common names, scientific names, eponyms, synonyms, and etymology (5 Volume Set)* (CRC press, 2012).

[17] Williams LAD, Caleb-Williams L (1997). Insecticidally active sesquiterpene furan from *Bontia daphnoides* L. Philipp. J. Sci..

[18] Adams, R.P., *Identification of essential oil Components by Gas Chromatography/Mass Spectrometry, 4th ed.*, USA. 804. (Allured Books, IL, 2009).

[19] Dong L-M (2018). Anti-MRSA sesquiterpenes from the semi-mangrove plant *Myoporum bontioides* A. Gray. Mar. Drugs..

[20] Bouzabata A (2022). HR-LC-ESI-Orbitrap-MS-based metabolic profiling coupled with chemometrics for the discrimination of different *Echinops spinosus* organs and evaluation of their antioxidant activity. Antioxidants..

[21] Indrayanto, G., G.S. Putra, and F. Suhud, *Chapter Six - Validation of in-vitro bioassay methods: Application in herbal drug research*, in *Profiles of Drug Substances, Excipients and Related Methodology* (ed. A.A. Al-Majed) 273–307 (Academic Press, 2021). 10.1016/bs.podrm.2020.07.005 33461699

[22] El-Halim SMA, Mamdouh MA, El-Haddad AE, Soliman SM (2020). Fabrication of anti-HSV-1 curcumin stabilized nanostructured proniosomal gel: molecular docking studies on thymidine kinase proteins. Sci. Pharm..

[23] Kant K, Lal UR, Kumar A, Ghosh M (2019). A merged molecular docking, ADME-T and dynamics approaches towards the genus of *Arisaema* as herpes simplex virus type 1 and type 2 inhibitors. Comput. Biol. Chem..

[24] Yoneda JD (2014). Docking of anti-HIV-1 oxoquinoline-acylhydrazone derivatives as potential HSV-1 DNA polymerase inhibitors. J. Mol. Struct..

[25] Bohn K, Zell R, Schacke M, Wutzler P, Sauerbrei A (2011). Gene polymorphism of thymidine kinase and DNA polymerase in clinical strains of herpes simplex virus. Antivir. Ther..

[26] Zardi-Bergaoui A, Jelizi S, Flamini G, Ascrizzi R, Ben Jannet H (2018). Comparative study of the chemical composition and bioactivities of essential oils of fresh and dry seeds from *Myoporum insulare* R. Br.. Ind. Crops Prod..

[27] Salama MTI (2017). Antimicrobial activity of essential oil of *Myoporum acuminatum* R. Br fruits, cultivated in Libya. J. Essent. Oil-Bear. Plants..

[28] Minh TT (2020). Chemical compositions and bioefficacy against Spodoptera litura of essential oil and ethyl acetate fraction from *Myoporum bontioides* leaves. Vietnam J. Chem..

[29] Youssef FS, Hamoud R, Ashour ML, Singab AN, Wink M (2014). Volatile oils from the aerial parts of *Eremophila maculata* and their antimicrobial activity. Chem. Biodivers..

[30] Sadgrove NJ, Hitchcock M, Watson K, Jones GL (2013). Chemical and biological characterization of novel essential oils from *Eremophila bignoniiflora* (F. Muell) (Myoporaceae): A traditional aboriginal Australian bush medicine. Phytother. Res..

[31] Thabet, A.A., Ayoub, I.M., Youssef, F.S., Al Sayed, E., & Singab, A.N.B. Essential oils from the leaves and flowers of *Leucophyllum frutescens* (Scrophulariaceae): phytochemical analysis and inhibitory effects against elastase and collagenase *in vitro. Nat. Prod. Res.*, 1–5 (2021). 10.1080/14786419.2021.2000981 34753359

[32] Yecheng D, Zhen Y, Yanzhen Y, Xiulian B (2008). Inhibitory activity against plant pathogenic fungi of extracts from *Myoporum bontioides* A. Gray and indentification of active ingredients. Pest Manag. Sci..

[33] Ayoub IM (2021). Anti-allergic, anti-inflammatory, and anti-hyperglycemic activity of *Chasmanthe aethiopica* leaf extract and its profiling using LC/MS and GLC/MS. Plants..

[34] Gad H, Al-Sayed E, Ayoub I (2021). Phytochemical discrimination of *Pinus* species based on GC–MS and ATR-IR analyses and their impact on *Helicobacter pylori*. Phytochem. Anal..

[35] Ashmawy AM, Ayoub IM, Eldahshan OA (2021). Chemical composition, cytotoxicity and molecular profiling of *Cordia africana* Lam. on human breast cancer cell line. Nat. Prod. Res..

[36] Younis MM (2022). GC/MS profiling, anti-collagenase, anti-elastase, anti-tyrosinase and anti-hyaluronidase activities of a *Stenocarpus sinuatu*s leaves extract. Plants..

[37] Korany DA, Ayoub IM, Labib RM, El-Ahmady SH, Singab ANB (2021). The impact of seasonal variation on the volatile profile of leaves and stems of *Brownea grandiceps* (Jacq.) with evaluation of their anti-mycobacterial and anti-inflammatory activities. S. Afr. J. Bot..

[38] Altyar AE, Ashour ML, Youssef FS (2020). *Premna odorata*: Seasonal metabolic variation in the essential oil composition of its leaf and verification of its anti-ageing potential *via in vitro* assays and molecular modelling. Biomolecules.

[39] Janibekov AA, Youssef FS, Ashour ML, Mamadalieva NZ (2018). New flavonoid glycosides from two *Astragalus* species (Fabaceae) and validation of their antihyperglycaemic activity using molecular modelling and *in vitro* studies. Ind. Crops Prod..

[40] Gamal El-Din MI, Youssef FS, Altyar AE, Ashour ML (2022). GC/MS analyses of the essential oils obtained from different *Jatropha* Species, Their discrimination using chemometric analysis and assessment of their antibacterial and anti-biofilm activities. Plants..

