# Supplementary material for: GC/MS profiling of essential oils from Bontia daphnoides L., chemometric discrimination, isolation of dehydroepingaione and evaluation of antiviral activity
Source: Sci Rep. 2022 Oct 21;12:17707. doi: 10.1038/s41598-022-22174-4 (PMC9587025; doi:10.1038/s41598-022-22174-4)
Supplement: Supplementary file 1 — Supplementary Information. [file 41598_2022_22174_MOESM1_ESM.pdf]

GC/MS Profiling of Essential oils from *Bontia daphnoides* L., Chemometric Discrimination, Isolation of Dehydroepingaione and Evaluation of Antiviral Activity

Amany A. Thabet[a], Saad Moghannem[b] , Iriny M. Ayoub[a], Fadia S. Youssef[a] , Eman Al Sayed[a] and Abdel Nasser B. Singab[a,c*]

[a] *Faculty of Pharmacy, Department of Pharmacognosy, Ain Shams University, 11566-Abbassia, Cairo, Egypt*

[b] *Department of Botany and Microbiology, Faculty of Science, Al-Azhar University, Nasr City, Cairo, Egypt*

[c] *Center for Drug Discovery Research and Development, Faculty of Pharmacy, Ain Shams University, Cairo 11566, Egypt*

Amany A. Thabet; E-mail: amany.thabet@pharma.asu.edu.eg; ORCID ID: 0000-0003-4388-0477

Saad Moghannem: E-mail: saadmoghannem@azhar.edu.eg; ORCID ID: 0000-0002-6686-5520

Iriny M. Ayoub; E-mail: irinyayoub@pharma.asu.edu.eg; ORCID ID: 0000-0003-2382-8241

Fadia S. Youssef; E-mail: fadiayoussef@pharma.asu.edu.eg; ORCID ID: 0000-0002-5871-2639

Eman Al Sayed; E-mail: em_alsayed@pharma.asu.edu.eg; ORCID ID: 0000-0001-7869-2009

***Corresponding author:**

Abdel Nasser B. Singab; E-mail: dean@pharma.asu.edu.eg; Tel: +2 02 2405 1120; Fax: +2 02 2405 1107; ORCID ID: 0000-0001-7445-963X

Legends

Table S1: ^{1}H- and ^{13}C-NMR spectroscopic data for dehydroepingaione.
Figure S1: (**a**) *B. daphnoides* leaves and stem, (**b**) *B. daphnoides* leaves, fruits and flowers, (**c**) *B. daphnoides* fruits and (**d**) and (**e**) *B. daphnoides* flowers.

Fig. S2a: ^{1}H NMR spectrum of dehydroepingaione.

Fig. S2b: APT spectrum of dehydroepingaione.

Fig. S2c: HSQC spectrum of dehydroepingaione.

Fig. S2d: COSY spectrum of dehydroepingaione.

Figure S3: Calibration curve of peak area vs. different concentrations of dehydroepingaione.

Figure S4: Effect of *B. daphnoides* leaves (BDL), *B. daphnoides* stems (BDS), *B. daphnoides* flowers, (BDF), *B. daphnoides* fruits (BDFR) and dehydroepingaione on Vero cells at different concentrations.

Table S1. ^{1}H and ^{13}C NMR spectroscopic data for dehydroepingaione

	δ_C	δ_H (Mult, Int), *J* in Hz
1	139.30	7.35 (*m*, 1H)
2	108.94	6.35 (*t*, 1H)
3	127.32	
4	72.75	4.91 (*m*, 1H)
5	33.42	2.22, 1.87 (*m*, 2H)
6	36.99	2.17, 1.87 (*m*, 2H)
7	82.32	
8	55.58	2.68 (*s*, 2H)
9	199.29	
10	125.51	6.14 (*m*, 1H)
11	155.01	
12	27.74	1.84 (*s*, 3H)
13	20.79	2.12 (*s*, 3H)
14	27.11	1.34 (*s*, 3H)
15	143.24	7.35 (*m*, 1H)

NMR data (δ) were measured ^{1}H NMR (400 MHz, $CDCl_3$) and ^{13}C NMR data (100 MHz, $CDCl_3$).

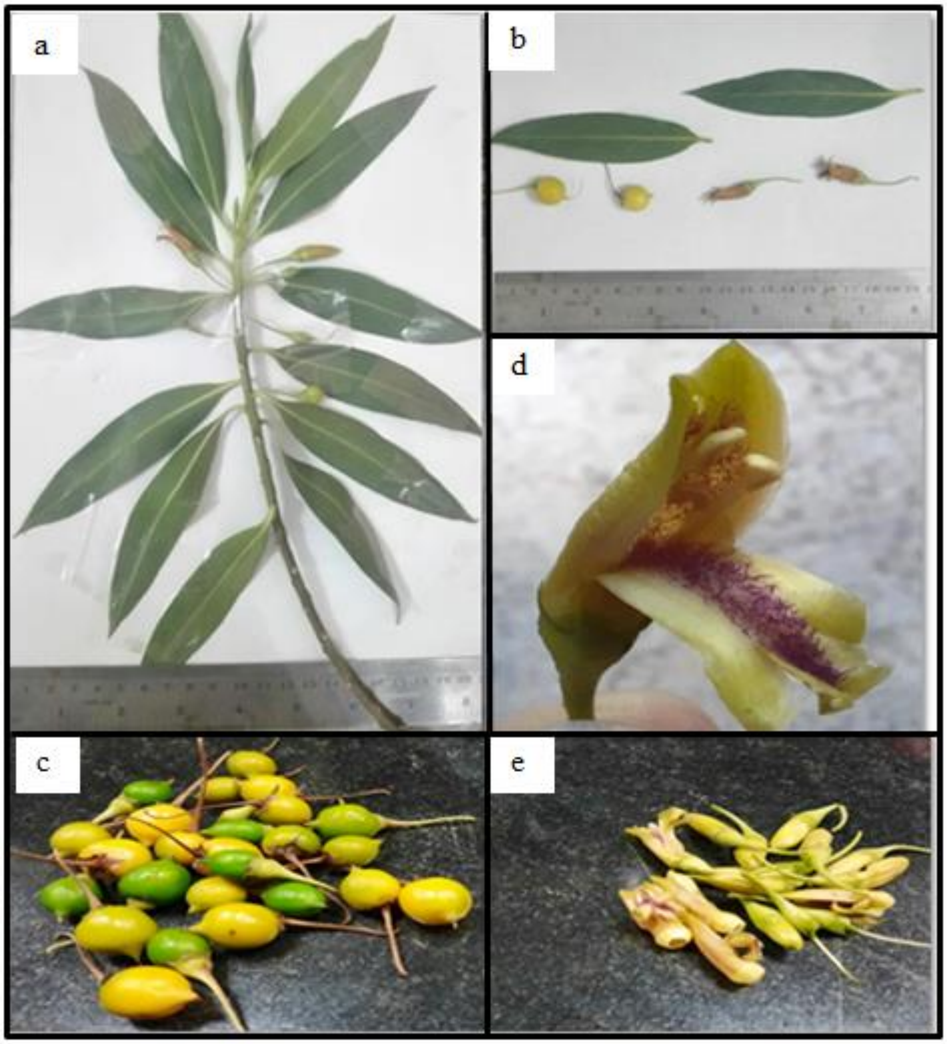

Figure S1: (**a**) *B. daphnoides* leaves and stem, (**b**) *B. daphnoides* leaves, fruits and flowers, (**c**) *B. daphnoides* fruits and (**d**) and (**e**) *B. daphnoides* flowers.

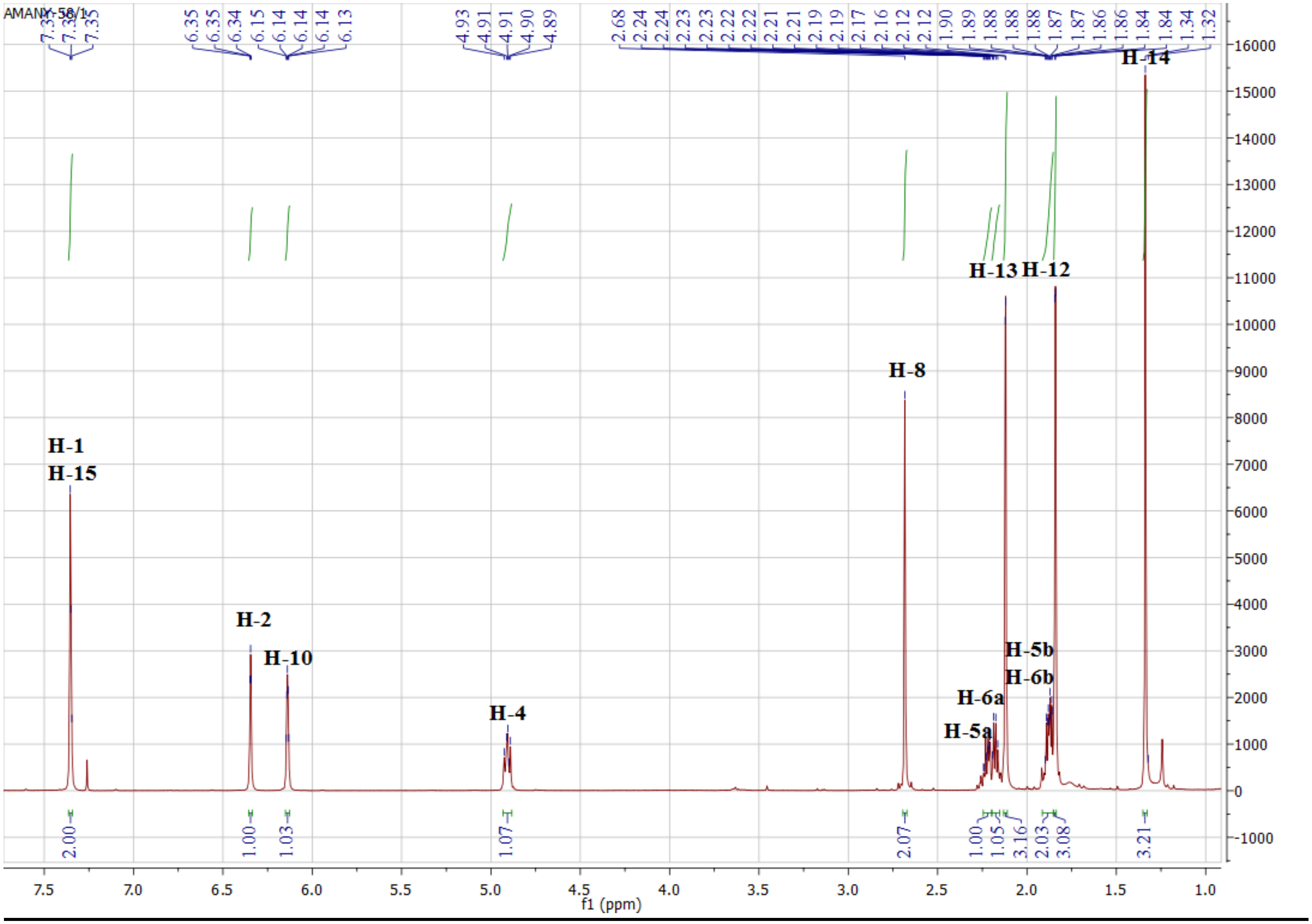

Fig. S2a: ^{1}H NMR spectrum of dehydroepingaione

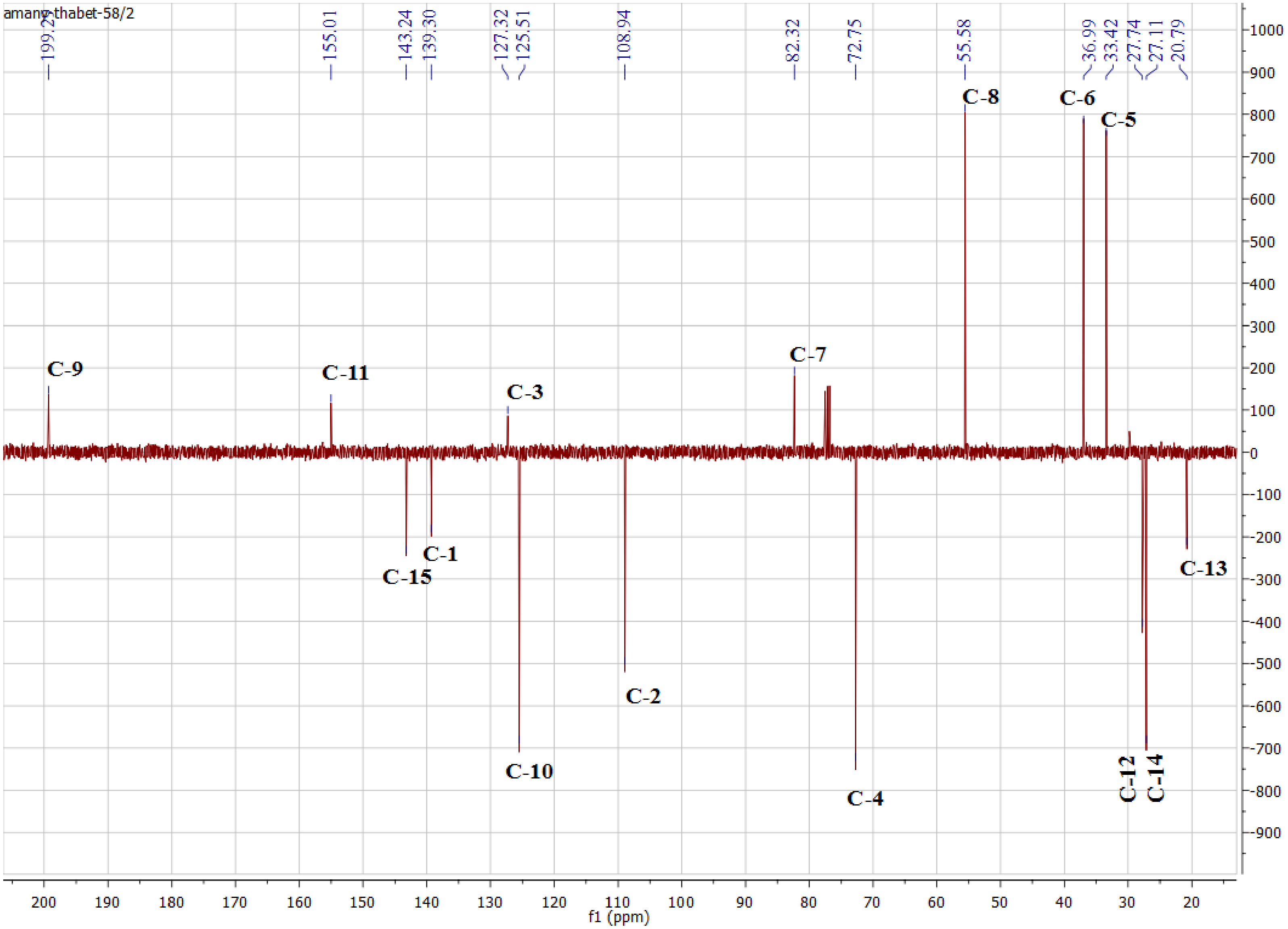

Fig. S2b: APT spectrum of dehydroepingaione

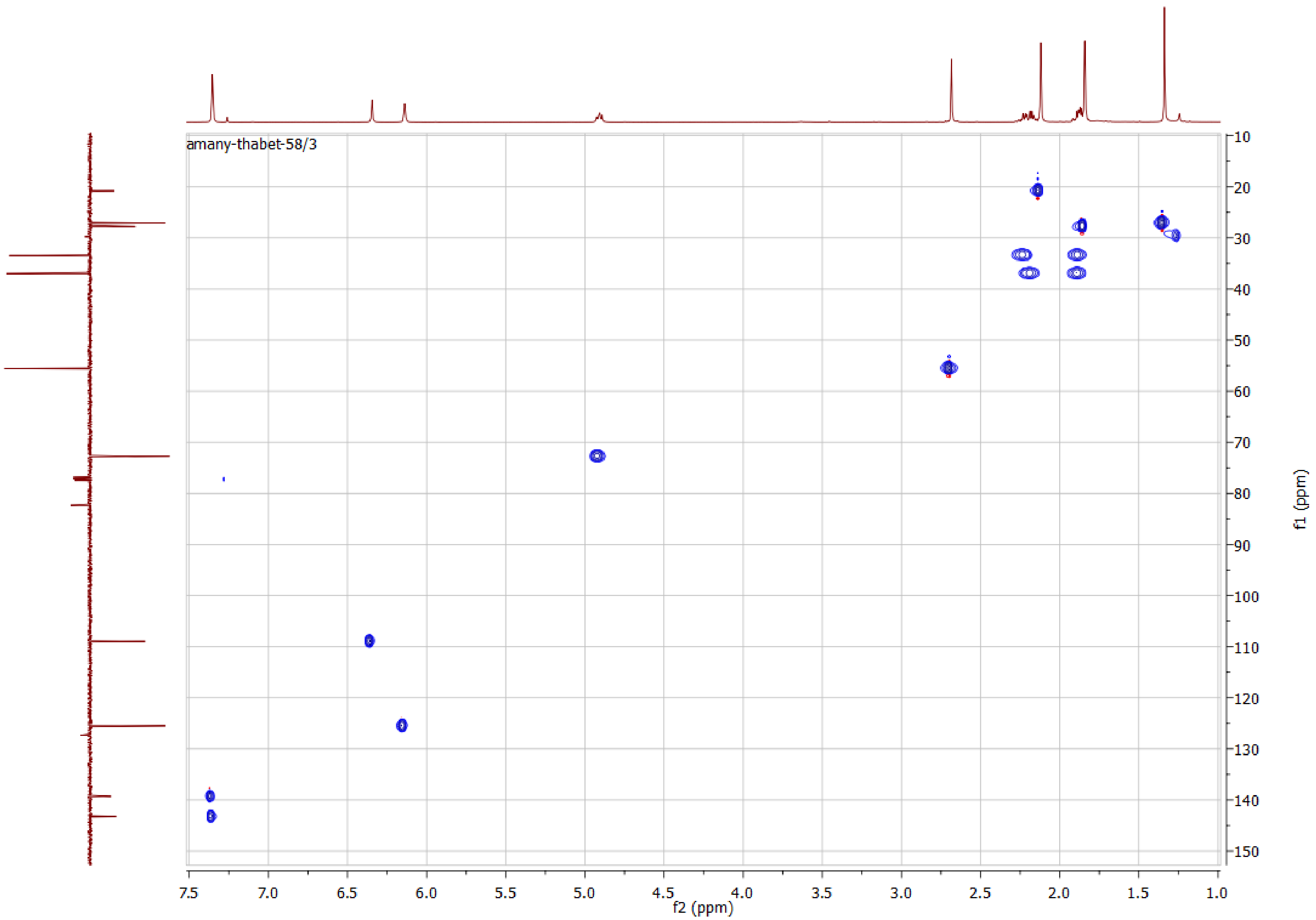

Fig. S2c: HSQC spectrum of dehydroepingaione

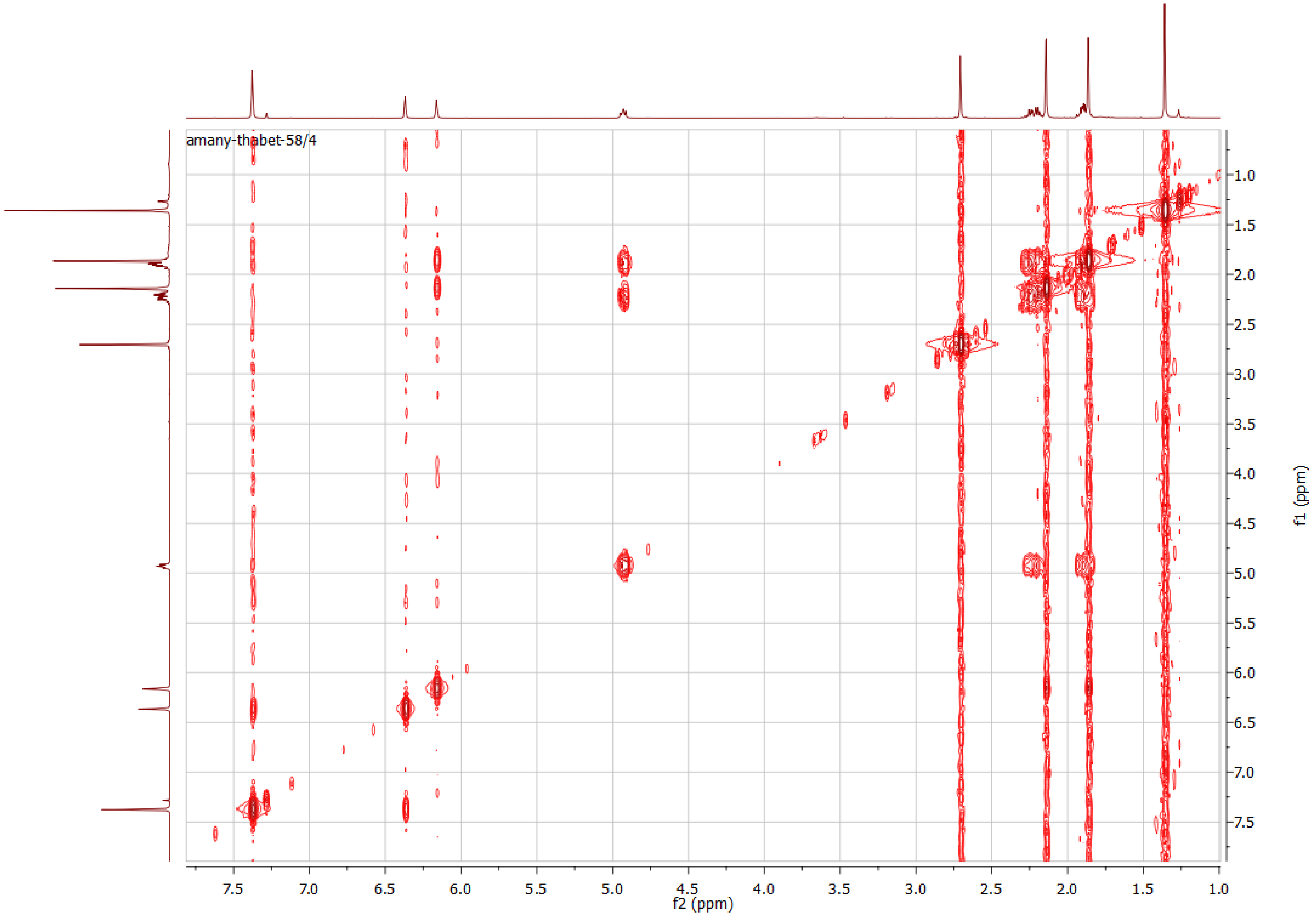

Fig. S2d: COSY spectrum of dehydroepingaione

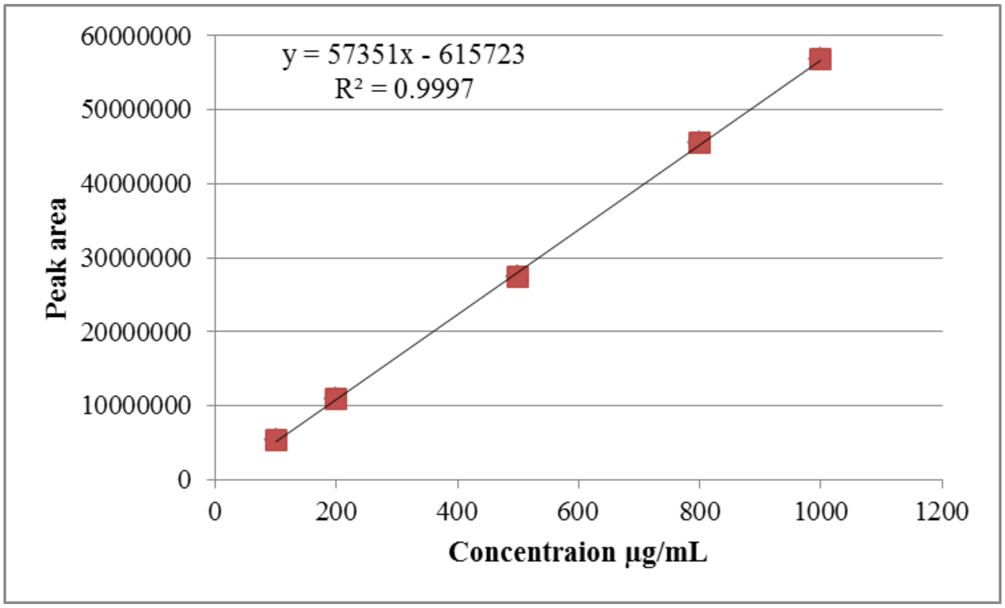

Figure S3: Calibration curve of peak area vs. different concentrations of dehydroepingaione.

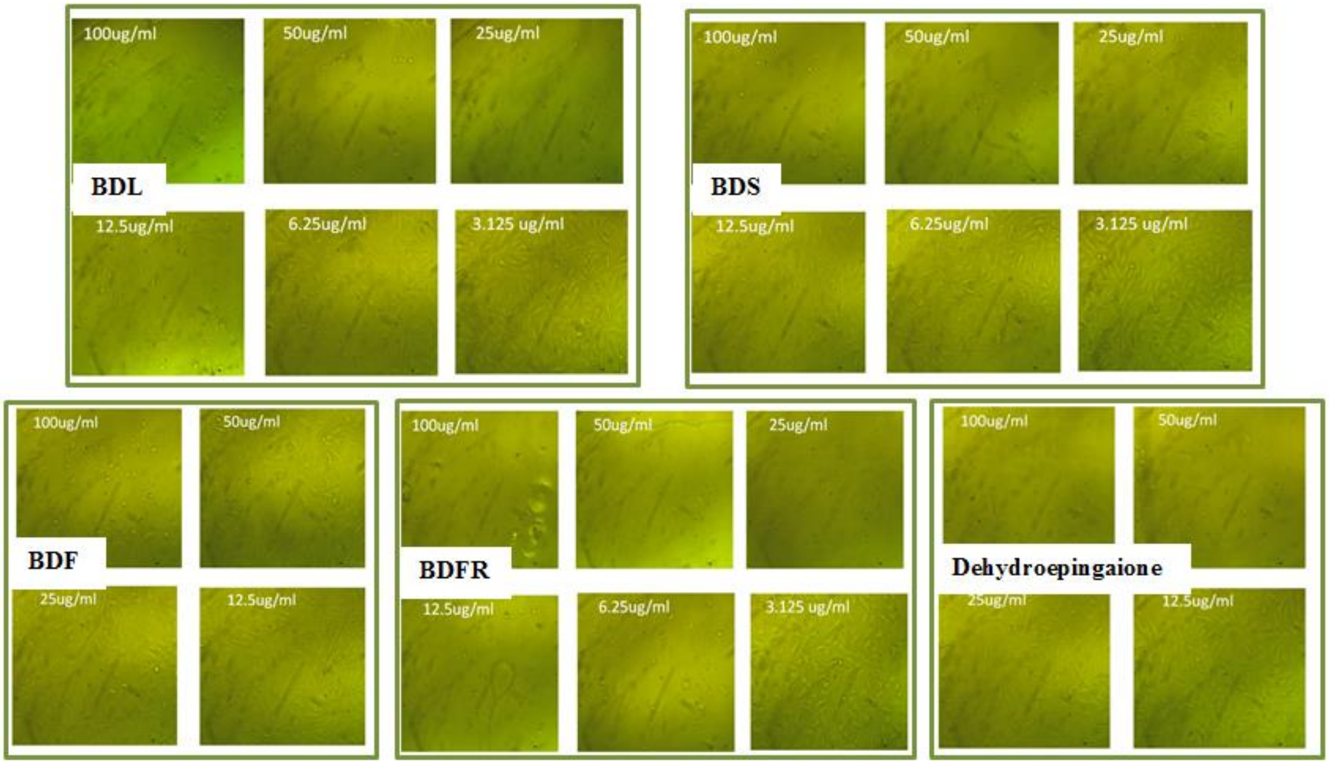

Figure S4: Effect of *B. daphnoides* leaves (BDL), *B. daphnoides* stems (BDS), *B. daphnoides* flowers, (BDF), *B. daphnoides* fruits (BDFR) and dehydroepingaione on Vero cells at different concentrations.
